# Beyond Kidney Function: A Neuro-Renal Perspective on Personalized Care in Pediatric Chronic Kidney Disease

**DOI:** 10.3390/medsci14050544

**Published:** 2026-09-03

**Authors:** Aarish Manzar, Una Tonkovic, Svetozar Mijuskovic, Aleksandar Sic, Aladin Altic, Jelena Pavlovic, Marko Baralic

**Affiliations:** 1 Department of Internal Medicine, Ziauddin Medical College, Karachi 75500, Pakistan; aarishsyedmanzar@gmail.com; 2 Faculty of Medicine, University of Belgrade, 11000 Belgrade, Serbia; unaunattt@gmail.com (U.T.); svetozarmijuskovic3@gmail.com (S.M.); jelena@pavlovic.rs (J.P.); 3 Department of Internal Medicine, Albert Einstein College of Medicine, Jacobi Medical Center/North Central Bronx Hospital, New York, NY 10467, USA; altica@nychhc.org; 4 Nephrology Clinic, University Clinical Center of Serbia, 11000 Belgrade, Serbia

**Keywords:** pediatric chronic kidney disease, neurodevelopment, kidney–brain axis, neuro-renal disorder, neurocognition, precision medicine approach

## Abstract

Pediatric chronic kidney disease (CKD) is traditionally viewed as a disorder of impaired renal function, yet its effects extend far beyond the kidneys. Growing evidence indicates that CKD may disrupt brain development through interconnected biological, vascular, inflammatory, and psychosocial mechanisms, with lasting consequences for cognition, mental health, and educational attainment. In this perspective, we propose the kidney-brain axis as a neuro-renal framework for understanding these neurological consequences of pediatric CKD. We discuss current evidence linking CKD with neurocognitive impairment, structural brain abnormalities, psychosocial burden, and adverse educational outcomes, while examining the biological mechanisms that may underlie these associations. We also consider the role of kidney transplantation, emphasizing that restoration of renal function does not necessarily restore normal neurodevelopmental trajectories. Finally, we outline the clinical implications of integrating routine neurodevelopmental assessment into pediatric nephrology care and highlight priorities for future research. Recognizing neurodevelopment as a core component of pediatric CKD may help redefine disease burden and support more comprehensive, lifelong care for affected children.

## 1. Introduction

Pediatric chronic kidney disease (CKD) has long been framed as a condition defined by its renal and uremic sequelae [1,2]. According to KDIGO, CKD is defined as abnormalities of kidney structure or function present for at least three months, with implications for health, and is classified according to cause, GFR category, and albuminuria category [3].

We propose a conceptual reframing in which pediatric CKD is viewed through a neuro-renal framework. Within this perspective, neurodevelopmental impairment is considered an important component of disease burden that extends beyond traditional renal manifestations [4,5,6].

Children living with CKD face an accumulation of chronic stressors, including strict dietary restrictions (low potassium, low phosphate regimens), persistent fear of disease exacerbation, immunosuppression and recurrent infections, which all collectively impose significant psychological and cognitive burdens during critical windows of neurodevelopment [7,8]. As medical advancements have improved long-term survival in pediatric CKD, the associated neuropsychiatric complications have become increasingly recognized. While the full impact of depression in pediatric CKD is still incompletely understood [9], studies in children with kidney failure have demonstrated lower adherence to treatment regimens and poorer health-related quality of life and adult CKD data suggest downstream consequences including earlier dialysis initiation, increased hospitalization and higher mortality, showing the urgency of recognizing and addressing mental health as another integral component of CKD care [10,11].

Improvements in pediatric nephrology have substantially increased survival and reduced many traditional CKD-related complications. As more children survive into adulthood, attention is increasingly shifting from survival alone toward long-term developmental, educational, and psychosocial outcomes. In this context, neurodevelopmental health may represent one of the most important yet underrecognized determinants of lifelong functioning among children with CKD.

## 2. The Kidney–Brain Axis: A Neuro-Renal Framework

We draw on the concept of the kidney–brain axis as the central organizing framework of this Perspective. In pediatric CKD, neurodevelopmental vulnerability is likely shaped by multiple interacting biological and clinical factors. Uremic and metabolic disturbances, including electrolyte and acid–base abnormalities, together with hypertension and disease severity, have been identified as potential contributors to neurological and neurocognitive dysfunction in children with CKD [12,13]. Sleep disturbance and fatigue may represent an additional component of this burden; data from the CKiD cohort demonstrated associations between lower measured GFR and greater weakness, low energy, and daytime sleepiness [14]. Anemia, impaired oxygen delivery, chronic inflammation, endocrine abnormalities, and vascular dysfunction may provide additional mechanistic links between kidney disease and the developing brain, although their individual contributions to neurodevelopmental outcomes remain incompletely defined [6,12,15]. Volume overload may further contribute through hypertension and hemodynamic instability, potentially disturbing cerebrovascular autoregulation and cerebral perfusion [12].

At the molecular level, the accumulation of uremic toxins provides another potential link between impaired kidney function and neurological dysfunction. Retained solutes such as indoxyl sulfate and guanidino compounds may exert direct and indirect neurotoxic effects through oxidative stress, neuroinflammation, endothelial and blood–brain barrier dysfunction, and altered neurotransmission [16]. Guanidino compounds, in particular, may disturb the balance between excitatory and inhibitory neurotransmission through activation of N-methyl-D-aspartate (NMDA) receptors and impairment of GABAergic signaling [17]. Uremic toxicity has also been implicated in peripheral neuropathy, illustrating that the neurological consequences of impaired renal clearance may extend beyond cognition [18]. However, much of this mechanistic evidence derives from experimental and adult CKD populations, and its specific contribution to the developing brain remains insufficiently defined. This further shows us the need for pediatric studies linking uremic toxin exposure with neurocognitive and neurodevelopmental outcomes.

These biological factors can further interact with repeated hospitalization, dialysis, transplantation, medication burden and disruption of education and everyday activities during critical periods of development [19]. Dialysis modality may also influence neurological risk in children with advanced CKD. Hemodialysis involves relatively rapid fluid, osmotic and blood pressure shifts that may affect cerebral function, whereas peritoneal dialysis provides more continuous fluid and solute removal. In both modalities, inadequate volume control and overhydration may contribute to hypertension and neurological complications, including encephalopathy [20,21].

Experimental and adult studies describing kidney-to-brain signaling, including α-synuclein propagation and the broader gut–kidney–brain axis, provide an indirect biological analogy for potential inter-organ communication between the kidney and brain [22,23,24]. However, these findings arise primarily from experimental and neurodegenerative settings and cannot be directly extrapolated to neurodevelopmental impairment in children with CKD. They should therefore be regarded as hypothesis-generating rather than evidence that renal dysfunction directly initiates neurological disease in pediatric CKD. The underlying etiology may further modify neurodevelopmental vulnerability. Children with CAKUT often develop CKD early in life, whereas glomerular diseases may present later and follow a more rapid or fluctuating course, with additional effects of inflammation, hypertension, proteinuria and immunosuppressive treatment. Neurodevelopmental risk may therefore differ across CKD etiologies.

We therefore propose that neurodevelopmental vulnerability should be regarded as an important dimension of pediatric CKD rather than solely as a late consequence of severe renal dysfunction. This does not imply that neurodevelopmental impairment is universal among children with CKD. Its presence and severity are heterogeneous and may vary according to age, CKD stage, underlying kidney disease, treatment modality, comorbidities and socioeconomic conditions. Within this framework, neurological, cognitive and psychological outcomes should therefore be considered alongside traditional nephrology metrics such as estimated glomerular filtration rate, proteinuria and blood pressure control when assessing the broader burden of disease.

## 3. Neurocognitive Consequences of Pediatric CKD

In pediatric CKD, cognitive dysfunction manifests as somewhat lower intellectual abilities with associated deficits in short-term memory, attention regulation and executive function, findings present across both preschool and school-age children [6]. Neuroimaging data point to structural and functional differences in the brain, with particular interest in the neurovascular system, though the underlying neurological mechanisms remain incompletely understood [6,25]. Hypertension is a potentially modifiable biomarker that may directly impact cognition, offering a concrete therapeutic target within this axis. However, our understanding of how these processes evolve in parallel with CKD progression is critically limited and longitudinal studies concurrently examining neurocognitive and neuroimaging outcomes are still lacking [6]. Analogous to other organ–brain interactions, a bidirectional relationship may plausibly exist in pediatric CKD: renal dysfunction and its downstream effects may adversely influence neurodevelopment, while neurocognitive and mental health difficulties may, in turn, affect disease self-management and treatment adherence, potentially contributing to poorer renal outcomes [6]. However, this proposed self-reinforcing cycle remains a conceptual hypothesis and has not yet been conclusively demonstrated in longitudinal pediatric CKD studies.

Neurocognitive impairment is one of the most consistently reported extra-renal complications of pediatric CKD. Although severe intellectual disability has become less common with advances in nephrology care, subtle but clinically meaningful deficits remain prevalent across multiple cognitive domains. Executive functioning, attention regulation, working memory, processing speed and academic achievement appear particularly vulnerable, even in children with mild-to-moderate disease stages [26,27].

Findings from the Chronic Kidney Disease in Children (CKiD) cohort further support this concept. Parent-reported assessments demonstrated disproportionately high rates of executive dysfunction, with at-risk scores observed in working memory, metacognition and planning domains, even among children with mild-to-moderate CKD. These findings suggest that clinically meaningful cognitive difficulties may be present despite the absence of severe neurological impairment [27].

These impairments appear to emerge early in the disease course and may progress alongside declining kidney function. The literature consistently links CKD with poorer neurodevelopmental outcomes across both cognitive and motor domains, suggesting that neurological involvement may begin long before the onset of kidney failure [4,28]. Neuroimaging findings further support this concept, demonstrating alterations in white matter integrity, cortical maturation and cerebellar development that collectively point toward widespread effects on the developing brain rather than isolated cognitive dysfunction. Emerging data also indicate that structural brain differences may already be present before dialysis or transplantation become necessary, with abnormal cerebellar growth trajectories associated with lower kidney function and executive dysfunction [29,30]. All of this challenges the traditional assumption that neurological complications arise primarily as late consequences of advanced renal disease and instead support the notion that neurodevelopmental disruption may represent an intrinsic component of pediatric CKD pathophysiology.

Cognitive dysfunction extends beyond formal neuropsychological testing. Deficits in attention, executive functioning and memory may directly affect classroom performance, learning capacity, organizational skills and long-term educational attainment [26,31]. low academic achievement was present in more than one-third of children with CKD, with mathematics representing one of the most consistently affected domains. These findings suggest that neurocognitive impairment may translate into measurable educational consequences long before kidney failure develops [32]. Academic difficulties observed in children with CKD likely reflect the combined effects of biological brain injury, chronic illness burden, treatment demands and psychosocial stressors rather than any single mechanism alone. Neurocognitive impairment in pediatric CKD should therefore be viewed as a heterogeneous profile of domain-specific difficulties and not a single uniform diagnosis.

## 4. The Psychosocial Burden of Growing Up with CKD

Beyond biological mechanisms, the neurodevelopmental burden of pediatric CKD is also shaped by social and educational disruption. Frequent medical appointments, recurrent hospitalizations, dialysis schedules, fatigue and physical limitations may interfere with school attendance, academic performance, peer relationships and participation in age-appropriate activities. During critical periods of cognitive and psychosocial development, these challenges can contribute to social isolation, reduced self-esteem, anxiety and difficulties in achieving developmental milestones [33,34,35].

The cumulative effect of these experiences may further amplify the neurocognitive vulnerabilities already associated with CKD-related physiological changes. Unlike many acute pediatric illnesses, CKD often requires children to adapt to long-term uncertainty regarding disease progression, transplantation and future health outcomes [36]. Geographic and socioeconomic disparities may further compound these challenges, as access to specialized pediatric nephrology and dialysis services often requires substantial travel to specialized pediatric dialysis centers which are often concentrated in tertiary hospitals, potentially disrupting: education, social participation and continuity of care. Children from more socioeconomically deprived backgrounds are more likely to require emergency dialysis initiation, reflecting disparities in access to timely nephrology care and pre-dialysis planning [37]. This sustained psychological burden develops during critical periods of identity formation, emotional maturation and social integration [36].

Children with CKD frequently experience disruptions in educational attainment that extend beyond temporary school absences. Recurrent hospitalizations, dialysis schedules, medical appointments and disease-related fatigue may reduce classroom participation and limit opportunities for social and academic development. Educational challenges occurring during sensitive periods of neurodevelopment may compound existing cognitive vulnerabilities, creating long-term consequences that extend into adolescence and adulthood. Peer relationships may be similarly affected. Visible manifestations of chronic illness, growth impairment, dietary restrictions and activity limitations may contribute to feelings of social difference and exclusion. Adolescence represents a particularly vulnerable period, as concerns regarding body image, autonomy and social belonging become increasingly important. For some patients, the psychological burden of living with CKD may therefore arise not only from the disease itself but also from the developmental experiences that the disease disrupts [33,36,38]. Arteriovenous fistulas used for hemodialysis may also affect body image during childhood and adolescence. Visible changes in the fistula-bearing arm may contribute to embarrassment, negative self-image, and feelings of difference or inferiority in relation to peers [39].

Viewed through a neurodevelopmental lens, these psychosocial stressors should not be considered separate from biological disease processes. Rather, they represent interacting exposures that may influence cognitive performance, emotional regulation, treatment adherence and overall developmental trajectories throughout childhood.

## 5. Kidney Transplantation and Neurodevelopment

Kidney transplantation is often regarded as the optimal treatment for pediatric kidney failure and may alleviate many of the metabolic and uremic disturbances implicated in neurocognitive dysfunction. However, transplantation should not be viewed as a complete reversal of neurodevelopmental risk. Cognitive deficits that emerge during early childhood may persist despite restoration of kidney function, while ongoing challenges related to immunosuppression, infection risk, medication burden and uncertainty regarding graft survival continue to affect psychological well-being [27,36,40]. Post-transplant corticosteroid-associated adverse effects may further influence psychosocial well-being. Weight gain, cushingoid appearance, acne and mood changes can be particularly distressing during adolescence, a developmental period in which body image and self-esteem are highly salient. These treatment-related changes may contribute to reduced self-confidence and social withdrawal despite successful restoration of kidney function [41,42]. These observations suggest that neurodevelopmental outcomes should be monitored longitudinally across the entire disease trajectory rather than only during periods of advanced renal impairment [27,36,40]. The transplantation process itself may also impose a considerable psychological burden. Prolonged waiting for a deceased-donor transplant can be associated with uncertainty, anxiety and emotional distress for both patients and families. This burden can be further intensified when a potential living-related donor is identified but subsequently excluded because of newly recognized medical contraindications, like hypertension or elevated urinary albumin excretion. For children and adolescents, the loss of a perceived opportunity for transplantation can represent a very distressing experience, reinforcing feelings of uncertainty of future health and treatment [43].

Kidney transplantation may improve selected cognitive and functional domains, potentially reflecting the correction of uremia, anemia, and other metabolic disturbances associated with advanced CKD. However, developmental trajectories do not consistently normalize after transplantation, particularly when neurocognitive deficits have developed before transplantation. Persistent difficulties may reflect the cumulative effects of pre-transplant disease, while post-transplant immunosuppression and treatment burden, together with educational disruption and psychosocial stress, may continue to influence cognitive and behavioral functioning. Age and developmental stage at transplantation may also be important, as prolonged exposure to CKD during sensitive periods of neurodevelopment could influence the extent of subsequent recovery. Kidney transplantation should therefore be viewed as an opportunity for neurocognitive improvement but not a guarantee of complete developmental normalization, supporting continued monitoring after transplantation [40,44,45].

Transplantation offers a unique opportunity to better understand the reversibility of neurodevelopmental injury in pediatric CKD. If cognitive deficits persist despite restoration of kidney function, this would suggest that at least part of the neurological burden develops early and may become biologically embedded. Conversely, substantial cognitive recovery following transplantation would support the existence of potentially modifiable disease mechanisms. Understanding this distinction represents one of the most important unanswered questions in pediatric neuro-nephrology.

## 6. Implications for Clinical Practice

The growing recognition of neurodevelopmental complications in pediatric CKD carries important implications for clinical care. Routine nephrology follow-up has focused on renal function, blood pressure control, growth parameters, metabolic abnormalities and preparation for kidney replacement therapy [46,47]. While these are essential treatment targets, they may fail to capture important dimensions of neurodevelopmental health.

Routine cognitive, behavioral and psychological assessment should be considered throughout the disease course, particularly during periods of rapid developmental change and major clinical transitions such as dialysis initiation, transplantation and transfer to adult care. Early identification of attention deficits, executive dysfunction, learning difficulties, anxiety or depressive symptoms may allow timely intervention before these challenges substantially affect educational achievement, treatment adherence and quality of life.

To translate this concept into clinical practice, we propose a structured but flexible framework for neurocognitive and psychosocial surveillance in children with CKD. Screening should cover core cognitive domains, including attention, executive function, memory, processing speed and academic functioning, together with behavioral symptoms, emotional well-being, fatigue, sleep and school participation. Brief screening may be considered at key clinical or developmental milestones, like CKD diagnosis, substantial deterioration in kidney function, initiation of dialysis, kidney transplantation, transition between school stages, adolescence, and transfer to adult care. Brief screening should be distinguished from formal neuropsychological assessment, which is more comprehensive and should be considered when screening identifies persistent or clinically significant difficulties, when there is unexplained academic decline, behavioral or emotional deterioration, poor treatment adherence potentially related to cognitive difficulties, or concern from the patient, family, school, or clinical team. Follow-up may be coordinated by the pediatric nephrology team, ideally with input from psychology, neuropsychology, psychiatry, developmental pediatrics, educational services and social work when indicated [12,13,14]. Personalized care here would mean adapting neurodevelopmental surveillance and support to each child according to developmental age, CKD stage and etiology, cognitive profile, dialysis or transplant status, educational needs and family and socioeconomic circumstances. These factors may guide the type and intensity of multidisciplinary follow-up rather than applying a uniform approach to all children with CKD. Patient-reported quality of life should complement cognitive and psychological screening, as it captures the broader impact of CKD on physical, emotional, social, and school functioning. Validated pediatric instruments, such as the Pediatric Quality of Life Inventory (PedsQL), can provide a structured assessment of these domains [48]. Such measures should complement, rather than replace, targeted neurocognitive and psychological assessment.

The proposed neuro-renal framework for clinical practice is summarized in Table 1. It integrates traditional nephrology assessment with neurodevelopmental screening, risk stratification, multidisciplinary intervention and long-term monitoring of both renal and neurodevelopmental outcomes. The framework is intended to support individualized assessment and follow-up rather than a uniform approach to all children with CKD. Importantly, it represents a proposed care framework rather than a validated clinical pathway, as optimal screening instruments, timing and intervals have not yet been established specifically for pediatric CKD populations.

Transition to adult nephrology care deserves particular attention within a neuro-renal framework. Adolescents with CKD must adapt to new healthcare systems while simultaneously navigating educational, social, and developmental challenges. Existing neurocognitive or psychosocial vulnerabilities may complicate this transition, reinforcing the need for continued neurodevelopmental monitoring beyond pediatric care. This period also frequently coincides with progression to advanced CKD and the initiation of dialysis or other forms of kidney replacement therapy. The need to adapt to a new healthcare environment while simultaneously managing the increasing treatment burden associated with end-stage kidney disease may pose additional challenges to adherence, self-management, and continuity of care, further supporting the need for structured and multidisciplinary transition programs. These concerns are supported by Crawford et al., who found that up to 50% of adolescents did not feel adequately prepared for transfer to adult care. Adolescents and their parents frequently reported anxiety related to leaving familiar pediatric services, while young adults described feeling overwhelmed by the adult care environment and out of place among older patients [49].

A multidisciplinary model involving pediatric nephrologists, neurologists, psychologists, psychiatrists, social workers, educational specialists and rehabilitation professionals may therefore provide a more comprehensive framework for care. Adopting a neuro-renal model would also require redefining how treatment success is measured. Preservation of kidney function is still essential [50], but optimal care should also seek to preserve cognitive development, educational attainment, emotional well-being and social participation. For children expected to survive decades with CKD or after transplantation, these outcomes may ultimately be as important as traditional renal endpoints.

## 7. Future Research Priorities

Despite growing awareness of neurocognitive complications in pediatric CKD, major knowledge gaps remain. Most available studies remain cross-sectional, involve relatively small cohorts and utilize heterogeneous neuropsychological and neuroimaging methodologies, limiting direct comparisons across populations.

Future studies should move beyond descriptive characterization of neurocognitive deficits and focus on identifying modifiable mechanisms. Particular attention should be directed toward the roles of hypertension, chronic inflammation, sleep disturbance, anemia and vascular dysfunction, all of which may represent potential therapeutic targets within the kidney-brain axis. An important next step will be the development and validation of standardized neurodevelopmental screening pathways for pediatric CKD. Similar to existing protocols for blood pressure monitoring and growth assessment, future care models could incorporate age-appropriate cognitive, behavioral and mental health evaluations throughout childhood and adolescence.

Several clinically relevant questions remain unanswered. Does aggressive blood pressure control preserve neurocognitive function? Can earlier transplantation improve long-term developmental trajectories? Which neuroimaging biomarkers best predict future cognitive impairment? Can psychological and educational interventions improve both developmental outcomes and treatment adherence? Addressing these questions will be critical for translating the neuro-renal framework into meaningful improvements in patient care.

## 8. Conclusions

Pediatric CKD should be recognized as a disorder that affects both kidney and brain development, with biological, cognitive, psychological, and social consequences that extend well beyond traditional measures of renal function. We propose a neuro-renal framework that places neurodevelopment alongside kidney function as a central determinant of long-term outcomes. This perspective supports a shift toward more personalized pediatric nephrology care, in which routine neurodevelopmental assessment, early identification of at-risk children, and multidisciplinary management become integral components of clinical practice rather than optional additions. Future research should determine how this framework can be translated into standardized care pathways and whether earlier neurodevelopmental interventions can improve both neurological and renal outcomes. Ultimately, preserving cognitive and psychosocial health may be as important as preserving kidney function in shaping lifelong outcomes for children living with CKD.

## Figures and Tables

**Table 1:** Proposed neuro-renal care framework for children with chronic kidney disease.

Component	Assessment/Domains
CKD Diagnosis	Entry point into the proposed neuro-renal care framework
Nephrology Assessment	eGFR; proteinuria; erythrocyturia; uACR (urine albumin/creatinine ratio); uPCR (urine protein/creatinine ratio); blood pressure; growth
Neurodevelopmental Screening	Cognition; mental health; school performance; social functioning
Risk Stratification	Integration of medical and neurodevelopmental factors
Multidisciplinary Intervention	Nephrology care; neurology/neuropsychology; psychology/mental health; educational support; social work/family support
Long-term Outcomes (Kidney + Brain)	Kidney: kidney function; transplant success; reduced complications. Brain/developmental: cognitive function; mental well-being; school and social achievement; quality of life

Abbreviations: CKD, chronic kidney disease; eGFR, estimated glomerular filtration rate; uACR, urinary albumin-to-creatinine ratio; uPCR, urinary protein-to-creatinine ratio.

## Data Availability

No new data were created or analyzed in this study. Data sharing is not applicable to this article.
